# Adolescent Aggression and Differentiation of Self: Guided Mindfulness Meditation in the Service of Individuation

**DOI:** 10.1100/tsw.2005.59

**Published:** 2005-06-23

**Authors:** Liora Birnbaum

**Affiliations:** Department of Social Work, Faculty of Social Sciences, College of Judea and Samaria, Ariel, Israel

**Keywords:** adolescence, aggression, differentiation, meditation, self-perception, Israel

## Abstract

This paper presents adolescent aggression as mediated by the level of differentiation of self. No research has directly addressed Bowen's notion that level of differentiation impacts child functioning including aggression. Level of differentiation is discussed in conjunction with social, gender and cultural norms as manifested in aggressive behavior. Female adolescent aggression is described as mainly relationship focused and expressed via verbal threats, intimidation and manipulation, while male aggression is described mainly as overt physical violence involving dominance and competitiveness. Research on differentiation focuses mainly on Western cultures that tend to be individualistic. Jewish-Israeli society is in transition from collectivistic to individualistic cultural values in the midst of ongoing hostilities. These processes create conflict regarding togetherness and individuality needs among adolescents, who are exposed to contradictory messages regarding separating and staying close. External as well as internal expressions of aggression (depression, suicide) are presented as coping strategies in the service of a wounded self-negotiating with the world. Guided mindfulness meditation is a powerful technique for facilitating healing and growth toward autonomy by helping adolescents connect to their inner voice. This technique may be especially useful in the adolescent search for self-awareness, meaning and life purpose. Bodily, cognitive and emotional experiences are treated as informative regarding the “self” and facilitate expansion of self-perception and individuality.

## INTRODUCTION

Studies on aggressive adolescents often deal with the level of influence various social systems such as family, friends, and school have on their behavior[1]. Research has repeatedly demonstrated that children's aggression is correlated with family functioning such as lack of appropriate parental monitoring of children, maladaptive parent-child interaction patterns, ineffective and inconsistent discipline, and the need to develop family problem-solving skills[2].

Togetherness and individuality are two major interrelated life forces central in family life. Structural family theory describes a family whose members are overly connected to one another as enmeshed. Indeed, adolescent enmeshment in reciprocal negativity in the family of origin is associated with aggressiveness and delinquency[3]. Other studies suggest the existence of low self-concept (developed in relation to others), low self-awareness, and high levels of frustration as associated with aggressive behavior[4]. However, no research has directly examined Bowen's theoretical concept, the differentiation of the self, in relation to adolescent aggression, or his notion that level of differentiation is linked to child functioning[5].

The literature to date on adolescent aggression contains little information illuminating the relationships between the process of separation-individuation[6], also known as the differentiation of the self[7], and aggressive behavior. Differentiation of the self is a developmental process where the child's growing "self" is constantly engaging with those of his parents, learning about closeness and distance as well as about the "self" in relation to others[8].

This article illuminates the significance of the differentiation of self as a fundamental variable mediating both external and internal aggressive behavior. Although neglected in the literature on adolescent aggression, this developmental process does emerge as significant in studies of youth suicide[9,10,11] and depression[12,13]. We intend to demonstrate that there is a link between aggression directed outward and aggression directed toward the self (as in suicide attempts), and that level of differentiation plays a mediating role in both. The relevance of the differentiation of self or self-autonomy as mediating aggressive behavior is dual in our view. First, it portrays the elements in the adolescent's "internal environment" that are acquired via interpersonal interactions and contribute to aggressive behavior. Second, it could shed new light on suicidal behavior among adolescents, which is the main cause of death in the age group of 15–19 years[9]. Both aspects play an important role in our suggested recommendations for educators and practitioners, which involve integrating a spiritual dimension into their work with adolescents. Mindfulness meditation is presented here as an intervention technique geared towards lowering negativity and facilitating individuality.

## LEVEL OF DIFFERENTIATION AND EXTERNAL AGGRESSION

Bowen[7] defined individuation or differentiation of self as the differentiation of emotional from intellectual functioning within the self and the degree of choice one has over what type of functioning will govern one's behavior. Family members are engaged in an ongoing dialog between cohesiveness and individuality, moving on the continuum between feeling similar and feeling unique. Low family differentiation often correlates with conflictual family relations, mainly due to the tendency to react emotionally. It is also often associated with confusing verbal and nonverbal parental messages directed to issues of dependency vs. autonomy. Generally speaking, the more differentiated the individual is, the more he is able to make choices based on his own personal goals and values. Bowen[7] suggests that there are three reactive tendencies in responding to emotion-evoking situations: withdrawal, counterattack, and defense of self. He does not discuss violence per se, but violence would appear to be an extreme form of the tendency to counterattack. For example, in a small pilot study, Bartle and Rosen[8] found that some adolescents and adults respond to emotional situations with their parents by slamming doors, throwing things, and becoming enraged. Thus, striking out at a person would also seem to be a possible response.

Birnbaum[14] studied the supervisory relationship between undergraduate social work students and their field supervisors. Supervisees with low levels of differentiation from their mothers reported more conflictual relationships with their supervisors, more verbal outbursts, and stronger negative feelings toward them. Poor differentiation of self can lead to fused relationships in which the distinct sense of "I" gets lost in the "we". Thus, violence may often serve as a distance-regulating mechanism, creating a "safe" distance and a temporary sense of individuality[8].

Research carried out with school-aged children demonstrates a clear relationship between low levels of differentiation and all of the following presenting problems: out-of-control behavior, misconduct behaviors, alcohol and drug dependence, lower academic achievement, and higher absenteeism[15]. The above problems are also strongly associated with violent behavior and with delinquency[16,17,18]. Aggressive behavior attached to low differentiation of self is mentioned in the psychopathology literature describing Munchausen's syndrome (MS). In MS, people may repeatedly harm themselves or fabricate fictitious medical emergencies in order to be admitted to hospital and obtain attention and affection from medical personnel[19]. In Munchausen's syndrome by proxy (MSBP), Meadow[in 19] identified an inverted form of the above syndrome. This involves the simulation or actual production of fictitious disease in children, mainly by their symbiotic mothers, who then appear as the "rescuing mother". Symptoms induced include bleeding, hematuria, and suffocation, and are often fatal. In both syndromes, the satisfaction of emotional needs through aggressive behavior involves either present or past symbiotic parent-child relationships. Moreover, child victims of MSBP apparently tend to exhibit MS as adolescents[19]. Despite such findings, the link proposed here between ongoing aggressive behavior and low differentiation has not been established directly in research. Nevertheless, while low level of differentiation does not always lead to aggression, we might benefit from considering its presence in cases of consistent aggressive behavior.

Aggression may become the language that certain adolescents use to negotiate with the world. Through it they are able to communicate needs and abilities. This language is familiar to children most disposed to develop low differentiation: those with a history of physical, emotional, or sexual abuse[20]. The inappropriate use made of the child's body, mind, or soul gives rise to the experience of penetration into a private space that is experienced as aggression. The child may learn to communicate with his surroundings through aggression, whether by verbal, physical, or sexual means. Children who exhibit aggressive behavior of any kind, including verbal aggression, are often simply doing what was done to them: using the object of their aggression to fulfill their own needs.

## DIFFERENTIATION OF SELF AND INTERNAL AGGRESSION

According to Stierlin[21], familial communication reflects the parent-child relationship system. When a symbiotic relationship exists in a family, communication is likely to include aggressive messages from parents to their children. These may involve power and dominance, tyranny, cognitive rigidity, hostility, confused messages, loss of boundaries, and dependency. Aggressive patterns of communication parallel the emotional experience on both sides, that of invasion of private space. Parents in symbiotic families often exhibit exploitive practical or emotional behavior towards their children. This is made possible since the system is operating under the assumption that security and satisfaction in life are achieved only within the family itself. The outside world is reflected by these parents as hostile and forbidding. They tend to develop a very narrow type of thinking in which the only way to function as a family is to tie their children closer to them. This is done by sabotaging the separation process at all costs. More specifically, the parent penetrates the teenager's private world and prevents normal development of separation-individuation[9]. In line with this, recommendations made by professionals in school violence prevention programs often involve creating and preserving a "private and safe space" for the child[22].

In his research regarding symbiotic parent-child relations, Richman[23] claims that the inability to proceed with the process of individuation and gaining self-autonomy is the main characteristic of adolescent suicide attempts. When adolescents' healthy need for autonomy meets unbearable closeness-oriented parental reactions, they may experience intense fear of losing their self-identity. This threatening closeness, in conjunction with hostile and alienating communication, contributes to the sense of an irresolvable problem and may encourage adolescent suicidal behavior as the ultimate means of achieving separation and autonomy. Freedom of the soul depends on destruction of the body.

Nahum-Sacks[9] examined the connection between familial symbiotic communication patterns, adolescents' mental state, and suicide attempts. She found that the most significant measure distinguishing between adolescents who did and did not attempt suicide is what she called “penetration into the autonomy”. While background factors did not directly predict suicide attempts, examination of the autonomy parameters showed a chain reaction. The higher the autonomy penetration perceived by the adolescent, the higher the levels of depression and suicide attempts. She found that 73% of the adolescents who attempted suicide in the research group experienced the level of penetration into their autonomy in their family as high. The above clearly indicates that level of family differentiation may involve aggressive behaviors on both sides: aggressive parental messages are experienced by the adolescents as an invasion of their private space. This learned aggression can be externalized in aggressive behavior towards others, or internalized and directed towards the self, turning into depression and suicidal behavior. The prevalence rate for major depression in adolescents has been estimated to range from 4 to 6.4% with the rate for females being about double that for males[24]. Recalling the hypothesized connection between depression and aggression directed at the “self”[25], it is worth noting that depression is common among adolescent girls with low levels of differentiation, and at times of emotional distress when adjustment to new situations is required[12,13]. This will be further discussed below.

Metaphorically speaking, depression may be viewed as emotional suicide, since the depressed person basically shuts down emotionally and often withdraws from many aspects of life. Being depressed thus enables one to exhibit such emotional suicidal behavior and stay physically alive at the same time.

## DIFFERENTIATION OF SELF, GENDER, CULTURE, AND AGGRESSION

Both differentiation of self and aggression are languages related to children’s way of negotiating with the world, and portray the way in which they communicate to others. The difference between genders lies in the different ways boys and girls perceive their realities as emotional individuals seeking intimacy. Girls’ need to separate from a “me-like” image (mother) while being socialized to attend to the emotional needs of others fosters development of a “self-in-relation”[26]. Men, on the other hand, are socialized towards goal-oriented achievement, dominance, and competitiveness[27], and are encouraged to shift away from their mothers early on and become independent[26]. Wade[10] described suicide as a resolution of separation-individuation among female adolescents. The stronger the emotional tie, the more energy has to be invested by the adolescent in order to separate. It seems that adolescent girls with low differentiation of self tend to use internal aggression coping strategies: depression and suicide. According to the literature, girls attempt to kill themselves more frequently, but it is boys who succeed more often. When suicide appears to be the only way to separate, it seems that boys “make sure” the goal will be achieved, as they were socialized to do. They tend to use more aggressive and lethal means than girls, such as firearms and hanging[28]. Girls’ suicide attempts may, in their socialized relational context, more often represent the proverbial “cry for help”.

Female aggression is described in studies on adolescent school violence as mainly relationship focused, with expressions of verbal threats, intimidation, and manipulation**,** while male aggression is described mainly as overt physical violence, involving dominance and competitiveness[29].

Aside from gender, we need to consider cultural factors influencing the process of differentiation. One of Bowen’s basic concepts is Societal Emotional Process. Just as families experience the counterbalancing forces of individuality and togetherness, these forces are operating in societies as well. As societal anxiety increases, so does the pressure and activity of togetherness forces. This gradually erodes the level of differentiation in the society, which seems to compromise functioning based on individuality[30]. According to Dwairy[31], the vast majority of research on differentiation has been conducted in individualistic Western societies that fit Bowen’s description. Collectivistic cultures might engage in a somewhat different dialog between these two forces. He describes Bedouin Arab adolescents in Israel as more collectivistic: they tend to be group oriented, to be controlled by external rather than internal factors, and to emphasize roles and norms rather then personal attributions. For example, some Muslim cultures still practice what is known as “murder for the sake of family honor”. Women whose behavior is perceived as promiscuous are executed in order to preserve the collective's good reputation. There are also cultures that go through a stage of transition from collectivism to individualism[32]. Jewish culture tends generally in the collectivistic direction, with strong emphasis on family and community. This was reflected in Israeli society, which for many years relied on collectivistic principals associated with the need to survive and establish an independent state. However, local sociopolitical processes and exposure to universal processes of globalization seem to be changing this society and leading it into a state of transition toward individualism[32]. Jewish Israeli society today faces many issues with both uniting and splitting effects, including Jewish/Arab, religious/nonreligious, and left/right political conflict[33]. Based on experience with group psychotherapy in Israel, Weinberg[33] apparently concluded that, compared to other countries, the level of interpersonal tolerance of an Israeli group is quite low and the level of aggression quite high. He also notes the presence of permanent intrusion and breaking of personal boundaries. He tied this phenomenon to some psychological aspects typifying Israeli society such as the tendency to express aggression when feeling vulnerable and to hide weakness; both reflect a need to separate from the "Diaspora Jew" image. Such social norms and conflicts play an active role in the formation of adolescent identity, forcing Israeli youth to take an "I position" in the context of a complex "we position", while exposed to conflicting expectations. Mandatory army service has the potential of promoting differentiation since the adolescent is asked to assume adult-like roles early on, including the need to use force (army activity) in order to "separate" and assure safety. On the other hand, ongoing loss and bereavement and the fear for life resulting from the same situation might enhance exactly the opposite.

Thus, it seems that level of differentiation might serve as an organizing variable, reorganizing the literature on adolescent aggression in a different way. Physical, emotional, external, or internal aggression may be viewed as coping strategies used by a wounded self for the sake of survival.

Such an approach to the data may be useful to educators and practitioners who are open and willing to expand their therapeutic space and language to a somewhat hidden aspect of their clients' lives: their spirituality. Rather than focusing on aggressive behavior or other specific symptoms of low differentiation, we see the "wounded self" as both the means and the goal of intervention. The technique presented below is geared towards re-establishing individuality within an undifferentiated wounded self. It therefore must touch on issues revolving around distorted boundaries in relationships, negative emotions, low self-worth, and giving preference to meeting the needs of others. We have found that these issues can be dealt with by training young people to tune into what we call their "inner voice", "inner guidance", or "inner wisdom". Since adolescents tend to be preoccupied with identifying themselves as different and unique[2], helping them find their own inner voice seems appropriate. Tuning in to inner wisdom is a spiritual practice acquired through mindfulness meditation, also known as "opening up" or insight meditation.

## SPIRITUALITY AND WORK WITH THE WOUNDED SELF

It can generally be stated that spirituality is being incorporated more and more into psychotherapeutic work. Some people identify their spirituality as religion based while others do not. Spirituality is here conceptualized to express human longing for a sense of meaning, fulfillment, and connection with something bigger that transcends ordinary life experiences[34]. In her writing on spiritual resources in family therapy, Griffith[35] states that "expanding therapeutic space to include spirituality, means making the therapy space open enough for the most significant others to be included in the construction of meaning, even when that significant other may be the Other, who is known by many names, whom some call God"[35]. By doing so, the therapist signals to his clients that a sacred space could be created between them and that it could hold their varied spiritual experiences, common or uncommon, including their beliefs regarding the way this world functions.

Birnbaum and Birnbaum[36] collected and content-analyzed 30 responses containing spiritually relevant statements from a youth Internet "suicide website" in order to gain insight into spirituality and suicide. Participants were adolescents who had made at least one suicidal attempt, responding to the question: “What do you believe that a suicide attempt means on a spiritual plane?” Four major themes were identified based on their responses:

1. Low evaluations of the suicidal self
2. Belief in a higher power
3. Belief in an ongoing cycle of life and death
4. Perceived relationship with God

Responses demonstrated that spiritual language is out there and is being used among suicidal adolescents who naturally see connections between spirituality and death wishes. Finally, these young individuals appear to be in search of their personal God, that unique aspect of themselves that connects them to the world and to others.

Mindfulness meditation is an insight meditation. Most kinds of meditations are geared towards achieving inner peace, self-reflection, or self-relaxation through the quieting of the mind, but there is more to meditation than just closing one’s eyes. Meditation is the way to achieve *letting go*. In meditation, one lets go of the complex world outside in order to reach the serene inside. The effort is directed to developing a mind that is willing to let go of things, let go of burdens. This is more than an effort; this is an attitude, a movement of the mind towards clarity[37]. Through the constant noting of one’s bodily sensations as well as any mental activities like feelings, thoughts, and imagery, one can train the mind to be fully aware and present. Since the nature of the mind is to wander here and there, this practice allows one to understand correctly the nature of the psychophysical occurrences taking place in one’s body. Constant awareness of those psychophysical processes helps develop the self into an observer that can access a deeper level of consciousness[37]. This is where the person is directly in touch with authentic needs, desires, and values. Mindfulness meditation has proven effective in reducing psychological stress and preventing relapse in depressed patients[38,39,40]. Bedow and Murphy[41] found it helpful in increasing empathetic capability, decreasing tendencies to take on others’ negative emotions (a possible outcome of low differentiation), and improving coping skills. Robbins[42] implemented a short-term insight meditation called the Dream Catcher Meditation with American Indian adolescents, which helped them to express unconscious conflicts and facilitated differentiation and healthy mutuality.

Mindfulness awareness is based on adopting a nonjudgmental attitude towards personal experiences[37]. A nonjudgmental attitude means experiences are not to be judged as “good or bad”, “successful or unsuccessful”. We treat them all as informative regarding the self. As a therapeutic intervention, it is best if a trained professional guides clients through the meditation and assists in becoming aware of insights or internal messages received. We identify and implement four stages: (1) physical relaxation, (2) independent mindfulness meditation, (3) guided meditation calling on inner voice, and (4) connecting with inner voice. In stage three, the therapist uses guided imagery in order to facilitate an encounter between the meditator and his or her perceived image of inner voice/guidance. In the fourth stage, the meditator remains attuned to any sensory, verbal, or imaginative insights.

## MINDFULNESS MEDITATION, AGGRESSION, AND INDIVIDUALITY

In our experience, consistent practice of mindfulness meditation can reduce negativity and anger[37]. Attunement to one’s true guiding self can facilitate the choice of alternative, nonaggressive forms of communication. The following example succinctly illustrates the flow of work and the emergence of insights regarding the self in Julia, a bachelor-level social work student who participated in an insight meditation workshop. The full process of Julia learning to identify and listen to her inner voice via meditation will be described elsewhere. We will focus on the progress she was able to make during each session, while searching for her individuality, and trying specifically to develop control over negative feelings such as anger, hostility, and resentment.

## CASE STUDY

Julia voluntarily participated in an experiential four-session group mindfulness meditation workshop based on the model described above. Subsequent work with her was done individually due to her strong emotional reaction in the first meditation session. In a private discussion, she described a conflictual and complex relationship with her mother, accompanied by strong feelings of resentment and anger toward her. She felt her mother's image was "forcing" itself on her. Julia's mother left her family in Russia when Julia was 13 years old and immigrated to the U.S. with her lover, leaving Julia with a depressed father and a younger brother. Without family to help, Julia assumed the mother role until her marriage at age 20. The two women have exchanged only a few written words every now and then, yet Julia felt that her mother's image was overly present in her life. She described how she had to keep herself constantly busy so as to prevent her mother from intruding on her thoughts. It felt to her like a war situation.

At the beginning of each meditation session, Julia was asked to set a goal. She was encouraged to pay careful attention to all experiences associated with her inner voice (guidance) while meditating and then report to the facilitator whether new insights surfaced about the self. Based on the complicated background she presented, it was decided to add daily meditative homework followed by journal writing. A few self-affirmations were mutually selected, focusing on Julia's desirable relationships with her immediate family members for her daily meditations. In addition, she was asked to write her mother a daily letter in her journal expressing her real thoughts and feelings.

> **Session 1 (45 min):** Julia's goal was to re-establish contact with her inner voice after the initial group meditation. Julia reported bodily sensations of heaviness and pressure in the beginning phase, then being surrounded by a bright light and feeling energy of love and warmth. She experienced an internal knowledge about not being alone. Her reported insight was: "I don't need to cry anymore, I feel less alone and more protected."
>
> **Session 2 (45 min):** Julia's goal this time was to distance herself from her mother's image and regain emotional balance by inviting her to an "internal meeting" with her own inner voice. She saw her mother "behind bars, looking modest and quiet" listening to her carefully. An insight followed her lengthy monolog: "I'm very complicated with myself, but it's more bearable. If I give up on my anger, I'll need something else to feel instead. I can forgive her…There is joy in me, more strength."
>
> **Session 3 (45 min):** Surprisingly**,** Julia's attention shifted to her teacher, Susan, who teaches the intervention class. Julia claimed that Susan was "depriving her of her attention and not being intimate enough with her" and said she felt "anxious, angry, and unsafe" in class. The goal set was to "find a way to face Susan so she can see me." This meditation focused on breathing, finding inner peace, and tuning in for new knowledge. The insights reported revolved around how Julia played along all these years with her family's dynamics, accepting the role of the "giver" with no right to take. "My current lesson is to be in a situation of providing service, and learn how to negotiate boundaries between giving and taking… The way to Susan goes through mother…"
>
> **Session 4 (45 min):** Julia appeared restless and fearful; she felt her mother was intruding into her work with clients. She was having trouble concentrating in sessions with her elderly female client as well as expressing her feelings in supervision. She decided to seek advice from her inner voice. She was astonished to report the appearance of her own

> image at age 13, and explained that this was when mother left home. Young Julia's insights: "The road is only blocked in your imagination, the limitations are yours, you are knocking on an open door… I chose a new way but I'm afraid of the responsibility, and of self-fulfillment… I'm used to explaining difficulties in terms of the relationship with my mother. Separating from her means separating from this defense as well… I'll have to explain success or failure in terms of my 'self'."

A dialog between meditator/client and facilitator/therapist opens and ends each session: In the beginning, its purpose is to help the client define the goal of the meditation session while at the end, it is aimed at processing the new knowledge revealed. The framework of the work involves uncovering the significance behind *every occurrence* experienced while meditating, be it physical, mental, or emotional. The different steps of the meditation are then linked with relevant key therapeutic issues and are used to help clients express conflicts or attach meaning to them. For example, when Julia reported feeling "heaviness on her shoulders and pressure on her head", the therapist encouraged her to tune into the uncomfortable feeling and try to attach it intuitively to some cognition, emotion, or a dilemma. This bodily reaction soon revealed Julia's ambivalence about working on her relationship with her mother and how that was projected onto the therapist. She identified feeling this "heaviness" every time she saw the therapist in the beginning phase.

Facilitating attention to one's inner or guiding voice means accepting surfaced contents as valid and purposeful. This assumption leads to asking questions of a different nature, questions that are geared towards revealing the purpose behind their appearance rather then evaluating their nature. When Julia blamed her teacher of depriving and discriminating against her in class, she was asked: "Assuming significant encounters with people, like yours with Susan, are not accidental, but hold meaning, purpose, and potential lessons at a given time in life for you both — what would *your* lesson be?" Treating Susan as a "coach" rather than a vicious teacher demonstrates perhaps the most valuable principals behind meditative work: acceptance and nonjudgmental attitude towards life events. Once she realized the mother-daughter conflict was projected onto other relationships (teacher, client), Julia saw them as involved in helping her deal with a life lesson: dealing with anxiety and self-assertion vis-à-vis feminine figures. It became apparent that Julia simply learned to hush and ignore her intuitive inner voice and experienced it as "dangerous" while giving way to external voices, especially her mother's. This nonblaming attitude shifted Julia's self-perception from feeling a victim to feeling an equal individual who chooses to focus on *her own* reactions as the target of change. Equality and mutuality are of significance here since they engage people in thinking about their own role in someone else's life lesson, as well.

In a concluding session (5 weeks later), Julia reported being in the midst of a distancing process from her mother: "It's a long way to go…" Her self-awareness had grown tremendously: "The emotional complexity with mother fulfilled a need but also blocked me… I got used to it and it became a part of me. Observing from a distance helped in identifying my different parts… Spending time by myself isn't scary anymore… I'm open to new activities and met new people; I started a new project at work that scared me before… My harsh feelings towards my mother allowed me not to look at myself… I mind me and my needs… The internal dialog with my inner voice continued after meditation sessions ended, it taught me to focus on myself… This experience symbolizes a big step in my personal and professional life…" Julia declined referral at this time, but the possibility of turning for counseling in the future was left open.

## DISCUSSION

The developmental literature emphasizes the differentiation variable as mediating the process of identity formation in adolescence, but very seldom relates this process to aggressive behavior.

Communication in symbiotic or abusive families is often characterized by aggressive messages delivered from parents to their children, which entail penetration of the latter's private space.

Overprotective parental patterns as well as parents who live their lives through their children may also influence level of self-autonomy. Coming from a wounded self, external or internal aggression may be conceived as a learned language used by adolescents with low levels of differentiation to negotiate with their world in order to fulfill emotional needs and gain self-autonomy.

Consistent practice of meditation and tuning in to the inner voice with sensitive professional guidance may enhance the sense of a distinct "self" and awareness of its wishes, goals, and needs. Taken together, these may well lead to a different perception of self[43] and facilitate individuality. This technique provides a unique way of exploring and utilizing hidden parts of the self as personal resources and sources of resilience, while providing a sense of self-control and wholeness. Accessing this state is precisely what may provide the necessary potential for positive change. The images and insights along with the process of making them meaningful seem to allow the self to heal itself. As the meditator becomes efficient in differentiating between internal and external voices, more self-oriented attitudes and behaviors can emerge.

Insights seem to expand the way in which people perceive themselves; they may change their self-concept in a manner that is almost self-directive. The gradually expanding private space created in meditation may function in a similar way to that of Winnicott's "transitional object"[44], connecting between internal and external realities. The use of such intermediate space lies also at the base of other therapeutic techniques such as play therapy and zeolotherapy (the use of pets) due to their known importance for the child in the process of separation-individuation[6].

Critiques of systems theory mainly question its universality. Major issues include insufficient attention to gender-related issues; to the social, political, and economic conditions that contribute to family dysfunction; and failure to acknowledge the realities of the power relationships that characterize our patriarchal society[45]. Bowen[7] was blamed for his tendency to overvalue stereotypically male characteristics and ignore the process by which women define themselves. Leaving the feminine experience out means not realizing that identity formation for women is not a matter of whether or not they define themselves in the context of relationships, but how relationships are used in the process. The feminine self-in-relation, which equips women with emotional interpersonal skills, was not presented as a source of strength for the family, but as yet another way to continue viewing mothers as the source of pathology in the family[45]. These limitations should be considered in reference to our suggested connection between Bowen's main concept, differentiation of the self, and spirituality. Mindfulness meditation and differentiation might also appear as oppositional since the first seems to be an individualistic practice while the other occurs mainly within the family system. In our view, this is an illusionary conflict. Both processes involve continuous overt and covert dialogs between the person and the environment regarding individuality and togetherness, though they work in opposite directions. Systemic process involves learning occurring first in the family, then working its way inward, resulting in personal knowledge about the nature of relationship between self and others (level of differentiation). When engaged in an internal mindfulness dialog, as demonstrated in the case example, the product, a more defined sense of self, is externalized in the direction of family or larger environment. Both processes focus on the fine balance between two life forces: individuality and togetherness. Bowen's theory is based on the idea that these two forces are competing, implying that differentiation requires developing the capacity to overcome the togetherness need. Mindfulness meditation allows the persistent meditators to connect and find their unique place in a much larger context, the Universe, and at the same time experience themselves as separate beings. Being mindful means learning to recognize the true nature of one's authentic inner voice as detached from external voices. Therefore, such knowledge transcends limitations posed by the external environment.

Spirituality in a religious context may be especially important in the adolescent search for self-awareness, meaning, and life purpose, and systems theory may help frame the struggle that adolescents experience regarding spiritual identity as the self turns and connects to God[46].

The main goal of mindfulness meditation is the development of an observant self that learns about one's actions, thoughts, and feelings from a nonjudgmental perspective. Awareness of emotions or cognitions that are at the base of human actions and reactions is a major factor associated with the process of differentiation. Various issues present themselves regarding meditation as a therapeutic intervention, including: In what way are the therapist role and therapeutic relationship influenced by this method? Who is it best suited to and in what situations? Is there potential for harm? We attempt to briefly address some of these issues here.

It is strongly recommended that guiding professionals be connected to and familiar with the nature of inner guidance, as this might help them handle unexpected client reactions. Suicidal people, for instance, could easily mistake the voice of their wounded self as "inner guidance" urging them to carry out their suicidal plan. The therapist's own knowledge and experience would tell them that true inner guidance is exclusively loving and universally geared towards choices and coping strategies that promote life, not death.

Regarding client selection, this method may not suit people with active psychotic or dissociative states, since it is based on one's ability to listen to authentic messages. Also, people with high control needs may feel frustrated at not being able to relax or connect to deeper parts of themselves, although they could be encouraged to practice letting go. Like any other intervention, a professional evaluation is essential in order to determine the right timing of integrating this technique into work with clients.

As for the therapeutic relationship, a unique alliance may develop when therapist and client meditate together, allowing optimal dialog based on equality. Integrating insight meditation into therapeutic work is not, of course, limited to work with adolescents. It does, however, present a special way of working with the evolving self while enhancing mutuality with this complex age group, which poses a challenge to most therapists and educators.

Most of the research done in the area of mindfulness has examined its usefulness among adults. Very little has been written about the use of this technique with adolescents. Since the main advantages of mindfulness training include reduced stress, improved focusing skills, self-awareness, and emotion regulation, it seems suitable in dealing with the type of issues faced by adolescents. It is also the kind of technique that can be taught and practiced in a group setting and, therefore, integrated into various support or therapy groups. Researchers and clinicians should be encouraged to explore the possible application of this self-developing process in challenging life situations. Teaching young people mindfulness meditation provides them with an effective life skill, which in time can turn into a major resource. It also strengthens the notion that spirituality is an integral part of life and, as such, should not be excluded from the therapy room or from scientific inquiry. The current limited research in this domain restricts our ability to understand comprehensively the full richness and variety of multidimensional human experience. The case example presented here illuminates one single highlighted process, but our ability to generalize is constrained by its specifics: the family dynamics, the participant's level of openness to spirituality and meditation, her relationship with the facilitator, etc. Further qualitative and quantitative research is needed in order to assess the relationship suggested by the literature, between level of differentiation and adolescent aggression.

## BIOSKETCH

**Liora Birnbaum, Ph.D.,** is a Certified Family Therapist. She is assistant chairperson of the Department of Social Work, Faculty of Social Sciences and director of the BA program in social work at the College of Judea and Samaria, Ariel, where she teaches clinical courses. She maintains a private psychotherapy practice in Kfar Yonah, Israel. E-mail: labirnbaum@bezeqint.net Website: http://www.yosh.ac.il/socialwork/Faculty.asp?n=Birnbaum_L
